# Implementation of tobacco control policy at the local level in Thailand: Performance evaluation and associated factors

**DOI:** 10.18332/tid/203868

**Published:** 2025-05-07

**Authors:** Chakkraphan Phetphum, Orawan Keeratisiroj, Artittaya Wangwonsin, Wutthichai Jariya

**Affiliations:** 1 Department of Community Health, Faculty of Public Health, Naresuan University, Phitsanulok, Thailand; 2 Tobacco Control Research Unit, Naresuan University, Phitsanulok, Thailand

**Keywords:** WHO FCTC, policy evaluation, law enforcement, policy implementation, tobacco policy

## Abstract

**INTRODUCTION:**

Tobacco use is one of the top five risk factors for disability-adjusted life years globally, including Thailand. Consequently, Thailand has enacted the Tobacco Products Control Act in alignment with the World Health Organization Framework Convention on Tobacco Control. To facilitate national tobacco control policies at the local level, Provincial Tobacco Products Control Committees (PTPCCs) have been established in all 77 provinces. This study aimed to assess the performance of PTPCCs in implementing the tobacco control policy and to identify its associated factors.

**METHODS:**

This cross-sectional survey utilized a self-reported online questionnaire to collect data between May and August 2023. The target population comprised secretaries of PTPCCs across 77 provinces. The survey achieved a completion rate of 75.32%, with responses from 58 provinces.

**RESULTS:**

Among the 58 responding provinces, 53% achieved the law enforcement indicator, 43% met the target for prevention of new smokers, and only 34% achieved the smoking cessation performance. Multiple logistic regression analysis revealed that adequate access to policy information was strongly associated with achieving law enforcement (AOR=10.53; 95% CI: 1.20–92.23). The availability of adequate media resources was significantly associated with successful prevention of new smokers (AOR=8.64; 95% CI: 2.09–35.67). Stronger characteristics of implementing agencies – referring to the provincial governor’s commitment to tobacco control policy and the engagement of public and private stakeholders – were positively associated with smoking cessation performance (AOR=2.05; 95% CI: 1.13–3.71).

**CONCLUSIONS:**

PTPCCs should be strengthened through adequate access to policy information, sufficient media resources, and strong provincial leadership with multi-sectoral engagement, as these factors are critical to effective policy performance. Enhancing these components will improve the implementation of tobacco control policy at the local level.

## INTRODUCTION

Tobacco use was among the five leading risk factors for disability-adjusted life years (DALYs) in 109 countries and territories in 2015, with a rise from 88 regions in 1990^1^. In Thailand, although the overall tobacco use declined steadily from 23.0% in 2004 to 17.4% in 2021, it remains a major public health concern^2^. In 2021, tobacco use was the second-highest attributable risk factor for combined mortality and disability burden in Thailand and accounted for 9.4% of total DALYs^3^. In addition, Thailand faces challenges from the tactics of the tobacco industry, including the introduction and promotion of new tobacco and nicotine products such as electronic cigarettes (e-cigarettes), online marketing strategies, and policy interference^4^. The rise of e-cigarettes among youth is particularly alarming, with a 2021 survey revealing that 30.5% of current e-cigarette users in Thailand are aged 15–24 years^5^.

Thailand has made significant progress in tobacco control policies since adopting the World Health Organization Framework Convention on Tobacco Control (WHO FCTC) in 2004^6^. The country has implemented a comprehensive tobacco product control act aligned with the WHO FCTC framework and adopted advanced tax policies^7^. Furthermore, the National Tobacco Control Strategy, following the WHO FCTC guidelines, utilizes a top-down policy approach with a National Tobacco Control Committee guiding policy decisions. This committee facilitates implementation by cascading policies from the national to the local levels^5,7^. However, strengthening the readiness and capacity of provincial tobacco control committees, which are responsible for local implementation, remains crucial.

To address local implementation, Thailand established Provincial Tobacco Products Control Committees (PTPCCs) in all 77 provinces under the Tobacco Products Control Act (B.E. 2560)^8^. Each committee is chaired by the Provincial Governor and includes representatives from various provincial government sectors and appointed experts, such as those from the police, education, provincial public relations, and local administrative organizations. The Chiefs of the Provincial Public Health Offices in all 76 provinces and the Deputy Director of the Bangkok Metropolitan Administration Health Department serve as the committee secretaries^8^. PTPCCs play crucial roles in implementing national tobacco control policies at local level. Their main duties include enforcing tobacco control laws, preventing minors from accessing tobacco products, and providing treatment programs for tobacco users^8^. However, challenges hinder their optimal effectiveness. These include communication gaps between national and provincial agencies^7^, unclear local tobacco control action plans^9^, and the absence of coordinated systems for monitoring and evaluation^5^.

Policy implementation is the critical bridge that connects the aspirations of policy design to real-world impact. These steps include planning, policy formulation, and adoption^10^. Implementing policies can broadly be defined as the process of translating policy intentions into actions, which includes activities carried out by various groups to achieve the objectives specified in the policy being implemented^11^. When a policy is implemented according to the intentions of its designers, it is considered to have high fidelity in the implementation process. This, in turn, can positively influence the desired policy outcome^12^. Therefore, effective implementation of Thailand’s tobacco control policies at the local level is critical to achieving goals and reducing tobacco use in Thailand.

Conducting policy-relevant research is essential for mobilizing public opinion and advocating for governmental action^13^. However, limited research on local tobacco control implementation in Thailand has identified significant enforcement gaps, such as limited knowledge and experience among local officers^14^, inadequate enforcement of tobacco control laws^14,15^, minimal public awareness campaigns^14^, and ineffective prevention of new smokers^16,17^. These findings indicate that local implementation may not meet national goals and highlight the need for improvement.

Therefore, this study aimed to evaluate the performance of PTPCCs in implementing tobacco control policies at the local level and to identify the associated factors by utilizing the Van Meter and Van Horn^18^ framework for policy implementation, which identifies six key components critical for successful policy implementation. This framework is well-suited for analyzing top-down policies, and aligns with Thailand’s centralized tobacco control approach, which emphasizes administrative management at the national level.

## METHODS

### Study design and population

This cross-sectional survey was conducted between May and August 2023. In terms of study population, Thailand is administratively divided into 77 provinces, each with a PTPCC. In 76 provinces, the Chief of the Provincial Public Health Office serves as the secretary of the PTPCC. Bangkok, which is the capital city of Thailand and is designated as a special administrative area, does not have a Provincial Public Health Office. Instead, the Deputy Director of the Bangkok Metropolitan Administration Health Department assumes the equivalent role as secretary of Bangkok’s PTPCC. Consequently, the target population of this study comprised the secretaries of the PTPCCs across all 77 provinces in Thailand.

The secretaries of PTPCCs play a vital role in gathering information, coordinating inter-agency efforts, preparing action plans, and reporting on PTPCC operations. In this study, the secretaries were considered as official representatives providing information in a questionnaire on behalf of their respective provinces. Accordingly, the study does not assess individual-level perspectives but rather evaluates implementation at the provincial level.

Out of the 77 provinces invited, a total of 58 secretaries completed the survey, resulting in a high response rate of 75.32%^19^. The high response rate is likely attributable to the clearly defined target population – PTPCC secretaries – and the use of official letters containing a QR code linking directly to the online questionnaire. These letters were sent directly to the PTPCC secretaries, including the Chiefs of the Provincial Public Health Offices in all 76 provinces and the Deputy Director of the Bangkok Metropolitan Administration Health Department. The formal nature of communication may have increased the likelihood of response from the target population. Furthermore, the follow-up reminder email was sent during the data collection period (May–August 2023) to those who had not yet returned the completed questionnaire.

### Measures

A self-reported online questionnaire in Thai, developed by the research team via Google Forms, was used for data collection. To ensure content validity, three experts evaluated the questionnaire items’ alignment with the study’s operational definitions using the Index of Item-Objective Congruence (IOC). All items achieved IOC scores exceeding the acceptable threshold of 0.5 (range: 0.67–1.00). Following this, the reliability of the validated questionnaire was tested with 58 respondents, who constituted the population targeted in the research due to its limited size. Likert scale questions were analyzed using Cronbach’s alpha to determine confidence values, revealing a score of 0.920 for tobacco control implementation by PTPCCs. The self-reported online questionnaire consisted of three parts as follows.

### Characteristics of the target population

We assessed a combination of open-ended questions and checklist items, for a total of four items, including gender, age, education level, and duration of work in the tobacco control field.

### Policy implementation factors (independent variables)

We applied the policy implementation model of Van Meter and Van Horn^18^, which includes six components that can influence successful policy implementation. We collected data based on the perceptions and experiences of secretaries of PTPCCs. The questionnaire comprises 15 questions across six sections, with all sections (except the policy resources section) using a five-point rating scale measured ranging from one (least) to five (most), except for the policy resources section, which uses a binary scale (adequate/inadequate). The details of the six sections and their components are as follows.

*Policy objectives and standards*

This section assessed the overall knowledge and understanding of PTPCCs regarding: 1) the objectives of national tobacco control policies; and 2) the indicators of the success of national tobacco control policies. The total possible score ranges 2–10.

*Policy resources*

This section consists of four questions that evaluate the availability of resources critical for successful implementation. The questions included, 1) budget allocation, 2) access to media for tobacco control campaigns, 3) information for informed decision-making, and 4) sufficiently dedicated personnel. The answers to each question were analyzed separately.

*Communication and coordination*

This section consists of two questions: 1) the quality of communication and collaboration within PTPCCs themselves, and 2) the quality of communication and collaboration between PTPCCs and central agencies responsible for disseminating tobacco control policies. The total possible score ranges 2–10.

*Characteristics of implementing agencies*

This section consists of two questions that examined: 1) the governor’s commitment towards tobacco control policy, and 2) the opportunity for public and private agencies’ participation in policy implementation. The total possible score ranges 2–10.

*Social conditions*

This section consists of two questions assessing: 1) support for tobacco control efforts from civil society organizations, and 2) the general public’s interest in these activities within the province. The total possible score ranges 2–10.

*Implementors’ attitude*

This section consists of two questions assessing: 1) the willingness of PTPCCs to achieve the national tobacco control policy goals, and 2) the dedication of health officers at the district level to enforcing the tobacco control policy. The total possible score ranges 2–10.

### PTPCCs implementation performance (dependent variables)

Evaluation of tobacco control implementation by PTPCCs was based on reporting operational results on three issues and comparing the operational results of PTPCCs with the national tobacco control indicators. Three questions were rated on a dichotomous scale (1 = achieved, 0 = not achieved). Answers were analyzed separately for each question. The details of each question are as follows.

*Law enforcement*

This assessed whether PTPCCs enforced tobacco control laws based on received complaints in designated places such as public spaces, workplaces, and public transportation, in accordance with established indicators and plans.

*Prevention of new smokers*

This investigated whether PTPCCs supported schools in implementing smoke-free school measures as specified in the indicators and plans.

*Smoking cessation*

This evaluated whether PTPCCs conducted screening and referrals into the treatment system as specified in the indicators and plans.

### Data collection

To ensure ethical research practices, researchers obtained approval from the relevant authorities, including the Chiefs of the Provincial Public Health Offices in all 76 provinces and the Deputy Director of the Bangkok Metropolitan Administration Health Department. Permission requests included a project summary, the questionnaire itself, and ethical approval certification from a research ethics committee.

Respondents from the target population were recruited through official letters sent to these authorities. The letters explained the research objectives through a clear project summary and provided a copy of the questionnaire for respondents to review the format and content beforehand. The letters included a QR code linked directly to the online questionnaire.

The online questionnaire process began with an informed consent section explaining the research objectives and its significance to potential respondents. Respondents were then given the opportunity to voluntarily participate in the study. The questionnaire took approximately 35–60 minutes to complete. The responses were directly stored in a secure database managed by the researchers. Following data collection, researchers conducted initial checks to ensure data quality and completeness.

### Data analysis

Analyses were conducted using SPSS version 17.0 for Windows (SPSS Inc., Chicago, IL, USA). Descriptive statistics were employed to characterize the distribution of the variables. These statistics included frequency, percentage, mean and standard deviation (SD). Bar graphs were used to depict PTPCC implementation performance across different categories, and comparisons were tested using the z-test for differences in two proportions.

Chi-squared tests and independent samples t-tests were used to assess the associations between each policy implementation factor and PTPCCs implementation performance. Multiple logistic regression with backward Wald test variable selection was then employed to develop a multivariable model. The initial model included all nine independent variables that were examined in the univariable analysis. These variables were selected based on theoretical plausibility and prior evidence of their relevance to policy implementation performance. The backward elimination method was used to retain only statistically significant predictors in the final model. The results are presented as odds ratio (OR) and adjusted odds ratio (AOR) with 95% confidence interval (CI). All levels of significance were set at p<0.05. The Hosmer-Lemeshow test was used to assess the goodness-of-fit of the logistic regression model. A non-significant p>0.05 indicates that the model fits the data well.

## RESULTS

The study involved 58 respondents who completed the questionnaire. Most of them (60.3%) identified as female. On average, the respondents were aged 42.40 ± 10.63 years. Most had completed a Bachelor’s degree (66.1%). They had an average of 4.71 years of experience in the tobacco control field (Table 1).

**Table 1:** The general characteristics of the respondents (secretaries of PTPCCs) and the policy implementation factors (N=58)

*Characteristics and factors*	*n (%) or* *Mean ± SD*
**Sex**	
Male	23 (39.7)
Female	35 (60.3)
**Age** (years)	42.40 ± 10.63
21–30	11 (19.0)
31–40	14 (24.1)
41–50	16 (27.6)
51–60	17 (29.3)
**Education level^a^**	
Bachelor’s	37 (66.1)
Master’s	19 (33.9)
**Duration of work in tobacco control field** (years)	4.71 ± 6.01
1–10	53 (91.4)
11–20	2 (3.4)
21–30	3 (5.2)
**Policy objectives and standards^b,d^**	7.64 ± 1.22
**Policy resources**	
**Budget^e^**	
Adequate	46 (79.3)
Inadequate	12 (20.7)
**Media^e^**	
Adequate	36 (62.1)
Inadequate	22 (37.9)
**Information^e^**	
Adequate	47 (81.0)
Inadequate	11 (19.0)
**Personnel^e^**	
Adequate	28 (48.3)
Inadequate	30 (51.7)
**Communication and coordination^c,f^**	7.60 ± 1.18
**Characteristics of implementing agencies^c,g^**	7.55 ± 1.27
**Social conditions^b,h^**	6.41 ± 1.53
**Implementors’ attitude^b,i^**	7.69 ± 1.43

a Missing=2.

b Number of items=2, possible score range 2–10, score range 4–10.

c Number of items=2, possible score range 2–10, score range 5–10.

d Assessment of the objectives and indicators of national tobacco control policies.

e Assessment of the availability of resources essential for successful tobacco policy implementation

f Assessment of communication and coordination within PTPCCs and between PTPCCs and central agencies.

g Assessment of the governor’s commitment to tobacco control policy and opportunities for participation by public and private agencies.

h Assessment of support from civil society organizations and the general public’s interest.

i Assessment of PTPCC’s willingness to achieve the policy’s goals and the dedication of district-level health officers to policy enforcement. PTPCCs: Provincial Tobacco Products Control Committees.

The policy implementation factors in Table 1 illustrate that the average score for policy objectives and standards was 7.64 ± 1.22. Policy resources for information were deemed adequate by 81.0% of the respondents, highlighting their strong availability, whereas policy resources for personnel were considered adequate by only 48.3%. Communication and coordination scored 7.60 ± 1.18. The characteristics of implementing agencies received an average score of 7.55 ± 1.27. Social conditions had an average score of 6.41 ± 1.53. Implementors’ attitude received a score of 7.69 ± 1.43.

The analysis of the policy implementation factors is detailed in Table 2. Most items within each category presented high or moderately high levels. However, ‘Support from civil society organizations’ and ‘General public’s interest’ within social conditions, and ‘Opportunity for public and private agencies’ participation’ within characteristics of implementing agencies, were identified as moderate level.

**Table 2:** The policy implementation factors and items, by level (N=58)

*Factors and items*	*Level, n (%)*				
	*Highest*	*High*	*Moderate*	*Low*	*Lowest*
**Policy objectives and standard**					
Objective of national policies	9 (15.5)	33 (56.9)	15 (25.9)	1 (1.7)	0
Indicator of national policies	7 (12.1)	32 (55.2)	18 (31.0)	1 (1.7)	0
**Communication and collaboration**					
Within PTPCCs themselves	6 (10.3)	32 (55.2)	19 (32.8)	1 (1.7)	0
Between PTPCCs and central agencies	9 (15.5)	34 (58.6)	13 (22.4)	2 (3.4)	0
**Characteristics of implementing agencies**					
Governor’s commitment towards policy	10 (17.2)	35 (60.3)	11 (19.0)	2 (3.4)	0
Opportunity for public and private agencies’ participation	4 (6.9)	20 (34.5)	26 (44.8)	7 (12.1)	1 (1.7)
**Social conditions**					
Support from civil society organizations	9 (15.5)	16 (27.6)	21 (36.2)	9 (15.5)	3 (5.2)
General public’s interest	1 (1.7)	14 (24.1)	32 (55.2)	11 (19.0)	0
**Implementors’ attitude**					
PTPCCs’ willingness to achieve the policy goals	11 (19.0)	34 (58.6)	11 (19.0)	2 (3.4)	0
Dedication of health officers at district level to enforce the policy	10 (17.2)	28 (48.3)	16 (27.6)	4 (6.9)	0

PTPCCs: Provincial Tobacco Products Control Committees.

**Figure 1:**
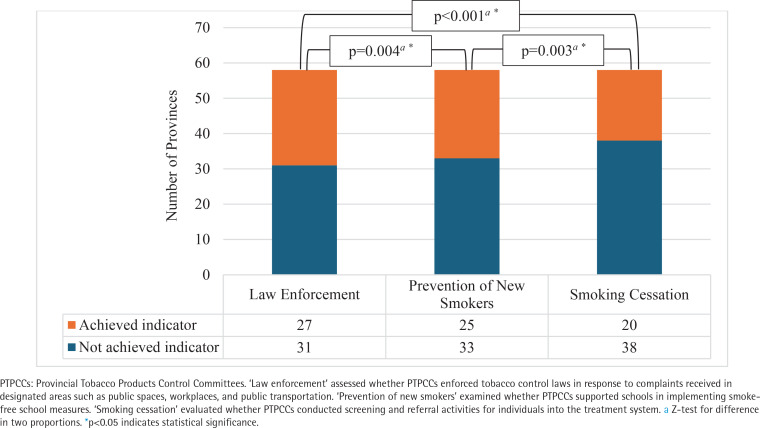
PTPCCs implementation performance (N=58)

The respondents assessed the implementation performance of PTPCCs in three key areas. The study found that 31 out of 58 provinces (53%) successfully fulfilled law enforcement, whereas the prevention of new smokers was achieved in 25 out of 58 provinces (43%). Smoking cessation was identified as the most challenging area, with only 20 out of 58 provinces (34%) meeting this indicator (Figure 1).

This study employed both univariable and multivariable analyses to examine the factors associated with the performance of PTPCCs implementation. The analysis was structured into three equation models, each corresponding to a different aspect of PTPCCs’ performance. The unadjusted analysis (Table 3) revealed significant associations (p<0.05) between several factors and specific performance outcomes. For law enforcement, five factors showed significant associations: policy objectives and standards (p=0.034), policy resources: information (p=0.006), policy resources: personnel (p=0.037), communication and coordination (p=0.016), and characteristics of implementing agencies (p=0.006). For the prevention of new smokers, significant associations were found with five factors: policy resources: media (p<0.001), policy resources: personnel (p=0.037), communication and coordination (p=0.013), characteristics of implementing agencies (p=0.010), and implementors’ attitude (p=0.028). For smoking cessation, two factors were significantly associated: policy objectives and standards (p=0.036) and implementors’ attitude (p=0.048).

**Table 3:** Univariable analysis of the associations between the policy implementation factors and PTPCCs implementation performance (N=58)

*Factors*	*Performance* *n (%) or Mean ± SD*		*p*
	*Achieved*	*Not achieved*	
**Law enforcement,** n	27	31	
Policy objectives and standards^a^	8.00 ± 1.14	7.32 ± 1.22	0.034^†^*
Policy resources: Budget (adequate)^b^	21 (77.8)	25 (80.6)	0.788^§^
Policy resources: Media (adequate)^b^	20 (74.1)	16 (51.6)	0.079^§^
Policy resources: Information (adequate)^b^	26 (96.3)	21 (67.7)	0.006^§^*
Policy resources: Personnel (adequate)^b^	17 (63.0)	11 (35.5)	0.037^§^*
Communication and coordination^c^	8.00 ± 1.14	7.26 ± 1.12	0.016^†^*
Characteristics of implementing agencies^d^	8.04 ± 1.22	7.13 ± 1.18	0.006^†^*
Social conditions^e^	6.52 ± 1.37	6.32 ± 1.68	0.632^†^
Implementors’ attitude^f^	8.04 ± 1.58	7.39 ± 1.23	0.084^†^
**Prevention of new smokers,** n	25	33	
Policy objectives and standards^a^	7.72 ± 1.14	7.58 ± 1.3	0.144^†^
Policy resources: Budget (adequate)^b^	20 (80.0)	26 (78.8)	0.910^§^
Policy resources: Media (adequate)^b^	22 (88.0)	14 (42.4)	<0.001^§^*
Policy resources: Information (adequate)^b^	23 (92.0)	24 (72.7)	0.064^§^
Policy resources: Personnel (adequate)^b^	16 (64.0)	12 (36.4)	0.037^§^*
Communication and coordination^c^	8.04 ± 1.17	7.27 ± 1.10	0.013^†^*
Characteristics of implementing agencies^d^	8.04 ± 1.31	7.18 ± 1.13	0.010^†^*
Social conditions^e^	6.80 ± 1.32	6.12 ± 1.63	0.095^†^
Implementors’ attitude^f^	8.16 ± 1.46	7.33 ± 1.31	0.028^†^*
**Smoking cessation,** n	20	38	
Policy objectives and standards^a^	8.10 ± 1.16	7.39 ± 2.00	0.036^†^*
Policy resources: budget (adequate)^b^	17 (85.0)	29 (76.3)	0.438^§^
Policy resources: media (adequate)^b^	13 (65.0)	23 (60.5)	0.739^§^
Policy resources: information (adequate)^b^	16 (80.0)	31 (81.6)	0.884^§^
Policy resources: personnel (adequate)^b^	10 (50.0)	18 (47.4)	0.849^§^
Communication and coordination^c^	7.70 ± 1.53	7.55 ± 0.98	0.698^†^
Characteristics of implementing agencies^d^	8.00 ± 1.49	7.32 ± 1.09	0.051^†^
Social conditions^e^	6.60 ± 1.73	6.32 ± 1.44	0.507^†^
Implementors’ attitude^f^	8.20 ± 1.44	7.42 ± 1.37	0.048^†^*

‘Law enforcement’ assessed whether PTPCCs enforced tobacco control laws in response to complaints received in designated areas such as public spaces, workplaces, and public transportation. ‘Prevention of new smokers’ examined whether PTPCCs supported schools in implementing smoke-free school measures. ‘Smoking cessation’ evaluated whether PTPCCs conducted screening and referral activities for individuals into the treatment system.

a Assessment of the objectives and indicators of national tobacco control policies.

b Assessment of the availability of resources essential for successful tobacco policy implementation.

c Assessment of communication and coordination within PTPCCs and between PTPCCs and central agencies.

d Assessment of the governor’s commitment to tobacco control policy and opportunities for participation by public and private agencies.

e Assessment of support from civil society organizations and the general public’s interest.

f Assessment of PTPCC’s willingness to achieve the policy’s goals and the dedication of district-level health officers to policy enforcement. PTPCCs: Provincial Tobacco Products Control Committees.

§ Chi-squared test.

† Independent t-test.

* p<0.05 indicates statistical significance.

Multivariable analysis (Table 4) revealed that, when controlling for communication and coordination, adequate policy resources for information were associated with a 10.53-fold (95% CI: 1.20–92.23) increase in the likelihood of achieving law enforcement performance compared to inadequate policy resources for information. When controlling for communication and coordination, adequate policy resources for media were linked to an 8.64-fold (95% CI: 2.09–35.67) increase in the likelihood of preventing new smokers compared to inadequate resources. When controlling policy resources for information, better characteristics of implementing agencies were associated with a 2.05-fold (95% CI: 1.13–3.71) increase in the likelihood of successful smoking cessation.

**Table 4:** Multivariable analysis of the associations between the policy implementation factors and PTPCCs implementation performance (N=58)

*Factors*	*Law enforcement*		*Prevention of new smokers*		*Smoking cessation*	
	*OR* *(95% CI)*	*AOR* *(95% CI)*	*OR* *(95% CI)*	*AOR* *(95% CI)*	*OR* *(95% CI)*	*AOR* *(95% CI)*
Policy resources: media^a,§^	-		9.95 (2.48–39.95)*	8.64 (2.09–35.67)*	-	
Policy resources: information^a,§^	12.38 (1.47–104.66)*	10.53 (1.20–92.23)*	-		0.90 (0.23–3.55)	0.24 (0.04–1.51)
Communication and coordination^b^	1.81 (1.09–3.03)*	1.73 (0.99–3.01)	1.85 (1.10–3.11)*	1.71 (0.96–3.03)	-	
Characteristics of implementing agencies^c^	-		-		1.59 (0.99–2.57)	2.05 (1.13–3.71)*
Hosmer-Lemeshow test		χ^2^=3.317 p=0.506		χ^2^=1.942 p=0.857		χ^2^=3.850 p=0.697

AOR: adjusted odds ratio; adjusted for all variables shown in each respective model.

§ Reference group is ‘Inadequate’ for all binary policy resource variables. ‘Not included’ indicates the variable was not entered into the model due to a lack of statistical significance in the univariable analysis or multicollinearity. ‘Law enforcement’ assessed whether PTPCCs enforced tobacco control laws in response to complaints received in designated areas such as public spaces, workplaces, and public transportation. ‘Prevention of new smokers’ examined whether PTPCCs supported schools in implementing smoke-free school measures. ‘Smoking cessation’ evaluated whether PTPCCs conducted screening and referral activities for individuals into the treatment system.

a Assessment of the availability of resources essential for successful tobacco policy implementation.

b Assessment of communication and coordination within PTPCCs and between PTPCCs and central agencies.

c Assessment of the governor’s commitment to tobacco control policy and opportunities for participation by public and private agencies. PTPCCs: Provincial Tobacco Products Control Committees.

* p<0.05 indicates statistical significance.

## DISCUSSION

This study represents a pioneering investigation into the performance and contributing factors of tobacco control implementation by PTPCCs in Thailand. The establishment of PTPCCs is crucial for coordinating and executing comprehensive tobacco control policies at both the provincial and local levels^8^. Their roles and responsibilities are consistent with findings from previous studies that underscore the importance of multi-sectoral coordination and stakeholder participation for the effective implementation of tobacco control measures^20-22^. However, the findings of this study indicate that there remain considerable opportunities for improvement across the three core areas of PTPCC performance: law enforcement, prevention of new smokers, and support for smoking cessation.

### Law enforcement

This study evaluated the effectiveness of PTPCCs in enforcing tobacco control laws by examining their response to complaints in designated areas, such as public spaces, workplaces, and public transportation. The law enforcement performance of PTPCCs is related primarily to WHO FCTC Articles 8 and 13^23^. This study’s results indicated that 31 out of 58 provinces (53%) successfully fulfilled the law enforcement performance of PTPCCs. This finding aligns with the 2016 smoke-free index by the South-East Asia Tobacco Control Alliance, which reviewed progress in 10 countries in the region, including Thailand^24^. The index showed that, while Thailand’s progress is commendable in most areas, law enforcement strategies remain an area in need of improvement^24^. Another study revealed that the implementation of Article 8 in Thailand was rated at 1.67 on a 0–3 scale, indicating less than effective enforcement^20^. Public understanding of smoke-free principles scored 1.28, and the effectiveness of smoke-free measures averaged 1.75. General smoke-free protection coverage was rated 1.98, but enforcement efforts scored only 1.0. Further protection in public places and private vehicles had mean ratings of 1.71 and 1.14, respectively^20^. The insufficient enforcement of tobacco laws aligns with findings from another study in Thailand, which indicated that tobacco vendors located near schools tend to violate retail laws, thereby increasing the risk of tobacco-related issues among young people^25^. Thus, relevant government agencies should stringently enforce regulations, specifically the prohibitions on selling individual cigarettes, displaying cigarettes openly at points of sale, and selling cigarettes to minors^15^. Government agencies involved should introduce initiatives to alter retailers’ attitudes and perceptions towards law compliance^15^.

The results of this study, derived from multiple analyses, highlighted that adequate information regarding tobacco policy was associated with a 10.53-fold increase in the likelihood of achieving law enforcement performance by PTPCCs, compared to having inadequate information (95% CI: 1.20–92.23). This finding aligns with the Van Meter and Van Horn^18^ framework that clear communication and well-informed implementers are vital to successful policy implementation. This may be because informed PTPCCs can interpret policies correctly, understand the legal framework and apply it appropriately in various contexts such as public spaces, workplaces, and public transportation. Access to comprehensive, accurate, and timely information about tobacco policies can provide support to enforce and implement the laws effectively^26^. Adequate information for achieving law enforcement underscores the importance of continuous education and training for leaders and partnerships^27^. Therefore, PTPCCs should invest in policy dissemination and capacity-building initiatives to enhance the overall effectiveness of tobacco control and enforcement.

### Prevention of new smokers

The performance of PTPCCs in preventing new smokers was examined and found to be achieved in 25 out of 58 provinces, representing approximately 43% of the total. This performance involves supporting schools in implementing smoke-free environments and is closely aligned with WHO FCTC Articles 12, 13, and 16^23^. The implementation of these efforts is evidenced by the expansion of school smoke-free policies to include e-cigarettes, which have been associated with increased legal knowledge, heightened harm perception, and a lower likelihood of current or future e-cigarette use among students in Thailand^28^. Implementing comprehensive school smoking policies, such as smoke-free school hours, along with teacher empowerment workshops, smoke-free campus programs, and policies, can contribute to preventing and reducing cigarette use among young people in high-risk school settings^29^. Thus, the proactive involvement of PTPCCs in these initiatives is crucial, as their support and enforcement capabilities can significantly enhance the effectiveness of smoke-free policies and programs. By continuing to collaborate with schools and other stakeholders, PTPCCs can further strengthen tobacco control efforts and ensure a comprehensive approach to preventing smoking initiation among youth.

The results of this study, derived from multiple analyses, indicated that the availability of adequate media resources was associated with an 8.64-fold increase in the likelihood of preventing new smokers, compared to having inadequate media resources (95% CI: 2.09–35.67). This finding underscores the critical role that the media play in public health initiatives, particularly in tobacco control efforts. According to the Van Meter and Van Horn^18^ framework, the availability and effective use of mass media resources can enhance visibility, acceptance, and support for policy implementation. Mass media interventions, including television, radio, newspapers, social media, billboards, posters, leaflets, and booklets, have the potential to reach and modify the knowledge, attitudes, and behavior of a large proportion of the community^30^. New media, such as social media and online platforms, play an active role in the prevention of smoking behavior and the intervention of smoking cessation behavior among adolescents^31^. Successful campaigns involve the implementation of combined school-based components, and the use of repetitive media messages delivered by multiple channels^30^. A previous study indicated that a considerable number of youths in Thailand are susceptible to smoking. Exposure to online cigarette advertising, less exposure to school anti-smoking education, and less exposure to anti-tobacco messages, were identified as being associated with increased smoking susceptibility among youths in Thailand^17^. Therefore, the effective utilization of media by PTPCCs to disseminate information and increase awareness of the risks associated with smoking is crucial for preventing the initiations of smoking in youth.

### Smoking cessation

The performance of PTPCCs in smoking cessation was examined in this study. This performance aligns with the WHO FCTC Article 14, which directly addresses measures for promoting smoking cessation and providing support for tobacco dependence treatment^23^. Thailand provides a wide range of smoking cessation services. The SMART Quit Clinic Program (FAH-SAI Clinics) is a government funded service launched in 2010, and operates in all 77 provinces with 552 clinics using the 5As model (Ask, Advise, Assess, Assist, Arrange)^32^. The FAH-SAI Clinics were found to be effective in assisting smokers in quitting smoking, with a continuous abstinence rate of 17.49% and 8.33% at 3 and 6 months^32^. Private sector services, particularly community pharmacies, have shown success with a 28.8% smoking abstinence rate^33^. The Thailand National Quitline (TNQ) offers cost-effective digital cessation support, with continuous abstinence rates up to 12 months and an average cost per quitter of $253^34^. It is evident that Thailand’s smoking cessation system offers a variety of support services. However, the results of this study found that the performance of PTPCCs in smoking cessation was identified as the most challenging, with only 20 out of 58 provinces (34%) achieving success. This might be that those smoking cessation services are primarily passive, meaning they rely on individuals who wish to quit smoking to initiate contact and seek help. It is essential for relevant agencies, such as mass media, to enhance awareness and promotion channels to ensure that those seeking to quit smoking are informed about and can access these services^35^. Additionally, there should be a development of proactive service models, such as community-based smoking cessation programs^21^. Therefore, PTPCCs should focus on increasing collaboration with community organizations, expanding media campaigns, and developing more proactive approaches to reach individuals who may not actively seek cessation support. These efforts could significantly enhance the success rate of smoking cessation programs across the country.

The findings from the multiple logistic regression analysis indicated that enhancing the characteristics of implementing agencies was associated with a 2.05-fold increase in the likelihood of successful smoking cessation (95% CI: 1.13–3.71). According to the Van Meter and Van Horn^18^ framework, leadership and commitment and stakeholder engagement are critical factors that determine how well a policy is executed and its eventual success. When the governor prioritizes tobacco control, it signals strong political will and leadership. This commitment can lead to better allocation of resources, increased attention to the issue, and stronger enforcement of tobacco control measures^12^. Involving a broad range of stakeholders, including public health organizations, community groups, and private sector partners, can enhance the reach and effectiveness of smoking cessation initiatives^36^. These stakeholders can contribute diverse perspectives, resources, and expertise, leading to more innovative and effective strategies^12^. Thus, PTPCCs should ensure strong leadership and commitment and foster stakeholder engagement to significantly improve their effectiveness in smoking cessation efforts.

### Strengths and limitations

This study’s nationwide scope, encompassing all 77 provinces of Thailand, provides a comprehensive overview of tobacco control policy implementation across diverse regions. This broad coverage enhances the generalizability and applicability of the findings to the entire country. Although the response rate was high, with participation from 58 provinces, the potential for non-response bias remains, as the 19 non-responding provinces may have different characteristics or face distinct implementation challenges. Furthermore, as this is the first study to investigate the PTPCCs’ performance and contributing factors in Thailand, the findings may have limited generalizability to other countries due to differing contextual and administrative structures.

The use of a self-administered questionnaire may cause both response bias and social desirability bias. Respondents may have felt compelled to provide overly positive responses due to concerns about potential negative consequences or a desire to portray their agencies in a favorable light. These biases could have led to an overestimation of both the PTPCCs’ implementation performance and the influencing factors.

The cross-sectional nature of the study means that it captures data at a single point in time, with independent and dependent variables collected simultaneously, thereby limiting the explanation of causal relationships. Therefore, continuous evaluation research or long-term studies are essential to confirm causality. Additionally, incorporating a qualitative approach in future studies can provide deeper insights into the underlying factors and contexts influencing the observed relationships, offering a more comprehensive understanding of the phenomena.

The small sample size (n=58) may have limited the statistical power of the multivariable analyses, resulting in wider confidence intervals and reduced precision of the estimated effects. In addition, although the analysis adjusted for important variables, the possibility of residual confounding – such as differences in region, staff size, or funding level – remains. These unmeasured factors may have influenced the observed associations. These limitations should be considered when interpreting the adjusted results. Future studies with larger sample sizes and more comprehensive factors may improve the stability and reliability of statistical estimates.

## CONCLUSIONS

This study evaluated the effectiveness of PTPCCs in Thailand, revealing key insights into their performance and areas for improvement. While PTPCCs have made some progress in enforcing tobacco control laws, preventing new smokers, and supporting smoking cessation, significant challenges remain. This study underscores the need for enhanced law enforcement strategies, more proactive initiatives to prevent youth smoking, and improved smoking cessation programs. To reinforce tobacco control efforts, PTPCCs should focus on ensuring that they have adequate resources and information for effective policy implementation, increasing media outreach, and strengthening their commitment and proactive engagement with stakeholders. Improving these components will be pivotal in advancing the effectiveness of Thailand’s tobacco control policies at the local implementation level.

## Data Availability

The data supporting this research are available from the authors on reasonable request.
